# Permanence, cost and mortality related to surgical admissions by the Unified Health System*

**DOI:** 10.1590/1518-8345.2618-3136

**Published:** 2019-04-29

**Authors:** Eduardo Rocha Covre, Willian Augusto de Melo, Maria Fernanda do Prado Tostes, Carlos Alexandre Molena Fernandes

**Affiliations:** 1 Universidade Estadual de Maringá, Maringá, PR, Brasil.; 2 Universidade Estadual do Paraná, Colegiado de Enfermagem, Paranavaí, PR, Brasil.

**Keywords:** Surgical Procedures, Operative, Time Series Studies, Hospitalization, Length of Stay, Costs and Cost Analysis, Mortality, Procedimentos Cirúrgicos Operatórios, Estudos de Séries Temporais, Hospitalização, Tempo de Internação, Custos e Análise de Custo, Mortalidade, Procedimientos Quirúrgicos Operativos, Estudios de Series Temporales, Hospitalización, Tiempo de Internación, Costos y Análisis de Costo, Mortalidad

## Abstract

**Objective:**

to analyze the time trend of surgical admissions by the Unified Health System according to hospital stay, costs and mortality by subgroups of surgical procedures in Brazil.

**Method:**

ecological study of time series. The variables surgical hospitalization, permanence, cost and mortality were obtained from the Department of Informatics of the Unified Health System. The trend analysis used the polynomial regression model.

**Results:**

in nine years, 37,565,785 surgical admissions were recorded. The mean duration of surgical admissions was constant (p = 0.449); the mean stay (3.8 days) was decreasing and significant (p <0.01); the mean cost (389.16 dollars) and mortality (1.63%) were increasing and significant (p <0.01). In subgroups of eye, thoracic, oncological and other surgeries, the temporal evolution of surgeries was increasing and significant (p <0.05). In contrast, endocrine glands, digestive tract, genitourinary, breast, reconstruction and buco-maxillofacial surgeries showed a significant trend of decline (p <0.05). In the other subgroups, the trend was constant.

**Conclusion:**

evidence shows the trend of surgical admissions in the last decade in the country and provide subsidies for the efficient elaboration of public policies, planning and management towards universal coverage in surgical care.

## Abstract (pt)

**Objetivo:**

analisar a tendência temporal das internações cirúrgicas pelo Sistema Único de Saúde segundo a permanência hospitalar, os custos e a mortalidade por subgrupos de procedimentos cirúrgicos no Brasil.

**Método:**

estudo ecológico de séries temporais. As variáveis internações cirúrgicas, permanência, custo e mortalidade foram obtidas do Departamento de Informática do Sistema Único de Saúde. A análise de tendência temporal utilizou o modelo de regressão polinomial.

**Resultados:**

em nove anos, 37.565.785 internações cirúrgicas foram registradas. A tendência temporal das internações cirúrgicas foi constante (p=0,449); a média de permanência (3,8 dias) foi decrescente e significativa (p<0,01); o custo médio (389,16 dólares) e mortalidade (1,63%) foram crescentes e significativos (p<0,01). Nos subgrupos de procedimentos cirúrgicos do aparelho da visão, torácica, cirurgia oncológica e outras cirurgias, a evolução temporal das cirurgias foi crescente e significativa (p<0,05). Em contraste, as cirurgias de glândulas endócrinas, aparelho digestivo, geniturinário, mama, reparadora e bucomaxilofacial apresentaram tendência significativa de declínio (p<0,05). Nos demais subgrupos, a tendência foi constante.

**Conclusão:**

as evidências geradas retratam a tendência das internações cirúrgicas na última década no país e fornecem subsídios para a elaboração eficiente de políticas públicas, planejamento e gestão na direção da cobertura universal em assistência cirúrgica.

## Abstract (es)

**Objetivo:**

analizar la tendencia temporal de las internaciones quirúrgicas por el Sistema Único de Salud según la permanencia hospitalaria, los costos y la mortalidad por subgrupos de procedimientos quirúrgicos en Brasil.

**Método:**

estudio ecológico de series temporales. Las variables internaciones quirúrgicas, permanencia, costo y mortalidad fueron obtenidas del Departamento de Informática del Sistema Único de Salud. El análisis de tendencia temporal utilizó el modelo de regresión polinomial.

**Resultados:**

en nueve años, 37.565.785 internaciones quirúrgicas fueron registradas. La tendencia temporal de las internaciones quirúrgicas fue constante (p=0,449), la media de permanencia (3,8 días) fue decreciente y significativa (p<0,01), el costo medio (389,16 dólares) y mortalidad (1,63%) fueron crecientes y significativos (p<0,01). En los subgrupos de procedimientos quirúrgicos del aparato de la visión, torácica, cirugía oncológica y otras cirugías, la evolución temporal de las cirugías fue creciente y significativa (p<0,05). En contraste, las cirugías de glándulas endócrinas, aparato digestivo, genitourinario, mama, reparadora y buco-maxilofacial presentaron tendencia significativa de declive (p<0,05). En los demás subgrupos, la tendencia fue constante.

**Conclusión:**

las evidencias generadas retratan la tendencia de las internaciones quirúrgicas en la última década en el país y fornece subsidios para la elaboración eficiente de políticas públicas, planeamiento y gestión en la dirección de la cobertura universal en asistencia quirúrgica.

## Introduction

Over the next 20 years, as a result of the epidemiological transition in many low- and middle-income countries, the need for surgery will increase continuously and substantially^(^ ^1^ ^)^. Estimated data on global surgical volume showed that 312.9 million surgical procedures occurred in 2012. Comparing this period with data from 2004, in eight years, there was a 38% increase in surgical volume, being more significant in countries with very low and low per capita expenditure in health, i.e., those who have invested $ 400 or less, per capita, in health care^(^ ^2^ ^)^.

Globally, in the last decade, investments in health systems have increased. Despite this, the effect of this investment on the volume of surgeries is little known^(^ ^3^ ^)^. In that direction, in 2013, a group of surgeons approached the editors of Lancet magazine to discuss surgery in the context of global public health, believing that the importance and role of surgeries in the health context were being neglected when compared to the other levels of health care^(^ ^4^ ^)^.

In response to this initiative, that magazine created the Lancet Commission on Global Surgery that same year. This Commission is composed of 25 members of a multidisciplinary team with collaborators in more than 110 countries with expertise in the areas of surgery, anesthesia, obstetrics, oncology, health policy, financing, economics and research. The group’s work is focused on assessing the current state of global surgical care and making concrete recommendations to improve access to surgery in order to achieve universal access to anesthetic and surgical care. The result of this work was the evaluation of surgical volume and production of an estimate of the need to perform 143 million additional surgical procedures per year to meet the global demand for surgery. In order to achieve this goal, at the global level, the Commission has set as a goal to be met by countries to perform 5,000 annual surgical procedures per 100,000 people by 2030^(^ ^4^ ^)^.

In Brazil, there is a shortage of available scientific evidence that discusses epidemiological data regarding surgeries that contemplate the different surgical specialties and their trend over the years^(^ ^5^ ^)^. In addition, scholars mention the scarcity of information on surgical volume in countries that can direct public policies to improve access to surgical care^(^ ^1^ ^)^.

In this sense, the present study was conducted to answer the following research question: In Brazil, in the last years, what is the time trend of surgical admissions by the Unified Health System according to the hospital stay, cost and mortality? It also aimed to fill a knowledge gap and produce information that helps in the reformulation of policies and the elaboration of complementary strategies to improve the surgical assistance in Brazil, as well as to analyze the temporal trend of surgical admissions by the Unified Health System according to hospital stay, costs and mortality by subgroups of surgical procedures in Brazil.

## Method

This is an ecological study of time series^(^ ^6^ ^)^ of the surgical procedures performed by the Brazilian Unified Health System (SUS) from 2008 to 2016. The data were obtained from the Department of Informatics of the Unified Health System (DATASUS)^(^ ^7^ ^)^.

Brazil is a country of continental dimensions, composed of five regions, which are subdivided into 26 states and one Federal District. It is the fifth most populous country in the world and the fifth in regional and social inequalities^(^ ^8^ ^-^ ^9^ ^)^. In relation to health, with the advent of SUS, the country became the largest in the world to have a public health system based on the principle of universality, equity and comprehensiveness^(^ ^10^ ^)^. It is estimated that the majority of the Brazilian population, approximately 80%, are SUS-dependent for actions related to health care^(^ ^11^ ^)^.

To obtain the data, access to DATASUS occurred in May 2017. The following variables were obtained: *i. Surgical Procedures*: these data were obtained through the admission variable, which corresponds to the number of authorizations for hospital admissions (AHA) approved in the period. The DATASUS system provides 16 subgroups of surgical procedures, namely minor surgeries and skin, subcutaneous tissue and mucosa surgery; endocrine gland surgery; central and peripheral nervous system surgery; surgery of the upper airways, face, head and neck; eye surgery; circulatory system surgery; digestive tract surgery, adnexal organs and abdominal wall; musculoskeletal system surgery; genitourinary system surgery; breast surgery; obstetric surgery; thoracic surgery; restorative surgery; buco-maxillofacial surgery; oncological surgery; and other surgeries.

Other surgeries include multiple surgeries (treatment in multiple surgeries), sequential surgeries (sequential procedures in postoperative bariatric surgery, neurosurgery, orthopedics, skull and buco-maxillofacial anomaly, oncology), surgical treatment in multiple trauma patients, and general surgical procedures (central vein catheterization by puncture, debridement of necrotizing fasciitis, ulcer/devitalized tissues debridement, ulcer/necrosis debridement, drainage of visceral/cavitary collections by catheterization). There is also the subgroup surgeries in nephrology; however, data for this category is not available. The surgeries that should be included in this subgroup are counted in the subgroup of surgeries of the genitourinary system. *ii. Average hospital stay* corresponds, in days, to the average length of hospital stay for the approved AHA, computed as admissions, in the said period. *iii. Average cost of admissions* corresponds to the total amount divided by the number of admissions. The monetary amounts were converted from reais to US dollars. US dollar quotation on 05/10/2018 (period of the research) = 3.54 reais. *iv. Mortality:* data were obtained by means of the variable mortality rate, which corresponds to the ratio between the number of deaths and the number of approved AHA, computed as admissions, in the research period, multiplied by the constant 100.

To obtain the variables, we accessed the Health Information (TABNET) on Health Care of the group of Hospital Production options (SIH/SUS). The chosen option was of consolidated data, by place of admission as of 2008, and the geographical scope chosen was Brazil, by region and federation unit.

The period from 2008 to 2016 was considered a temporal cut, since a similar pioneer study conducted previously in Brazil analyzed data from the period 1995 to 2007 available in DATASUS^(^ ^5^ ^)^ and brought relevant contributions on the subject. From this period, the temporal trend of surgical admissions was not investigated.

Through the “Procedures” option, the types of surgical procedures available from January 2008 to December 2016 were obtained. The “Content” option provided data on the number of admissions, mean length of stay, average cost of hospitalization and mortality rate due to surgical causes.

The time trend analysis was performed using polynomial regression models, considering that it has high statistical power and also because it is easier to formulate and interpret^(^ ^12^ ^)^. The polynomial model aims to find the curve that best fits the data, so as to describe the relationship between the dependent variable Y (surgical admission, length of stay, costs and surgical mortality), and the independent variable X (year of study). To deviate from the serial correlation between the terms of the regression equation, the variable year was centered in X-2012, since 2012 was the midpoint of the historical series.

As a measure of the accuracy of the model, the coefficient of determination was used (the closer R^2^ is to 1, the more adjusted the model is). Initially, the simple linear regression model (Y = β0 + β 1X) was tested and, then, those with higher orders, with second (Y = β0 + β1X + β2X2) or third degree (Y = β0 + β1X + β2X2 + β3X3). The best model was considered the one that presented the highest statistical significance (lower value of *p*) and residues without vices. When two models proved to be similar from the statistical point of view, for the same variable, the simplest model was chosen, taking into account the principle of parsimony. A trend was considered significant when the estimated model obtained p-value <0.05. Data tabulation and statistical analysis were performed by Microsoft Excel 2013 and R Software.

Because it is a study using data obtained from secondary sources, without identification of research subjects and whose access is in the public domain, there was no need of appreciation by the Ethics Committee for Research with Human Beings.

## Results

In Brazil, according to data from the SIH/SUS, 37,565,785 surgical admissions occurred between 2008 and 2016. Regarding the subgroup of surgical procedures, obstetric surgeries (8,583,315), digestive system surgeries (6,426,105 surgeries) and musculoskeletal surgeries (6,289,449) stood out.

In the nine-year period, the time evolution of surgical admissions was constant (p = 0.449). Regarding the subgroups, eye, thoracic, oncologic surgery and other surgeries showed a significant upward trend (p <0.05). In contrast, surgeries of endocrine glands, digestive tract, genitourinary, breast, reconstruction and buco-maxillofacial tissues showed a significant trend of decline (p <0.05). The others presented a constant trend, according to Table 1.

**Table 1:** Temporal trend of surgical admissions according to subgroup of procedures. Brazil, 2008 to 2016*

Subgroup^†^	Number of admissions	Model	R^2‡^	*p* ^§^	Trend
Minor surgeries	968,366	y=55.144+0.112x-0.140x^2^-0.035x^3^	0.235	0.185	Constant
Endocrine glands	115,413	y=6.669-0.042x-0.029x^2^-0.006x^3^	0.575	0.017	Decreasing
Nervous system	761,324	y=44.532-0.036x-0.288x^2^-0.015x^3^	0.090	0.432	Constant
Head and neck	1,181,851	y=70.534+2.545x-0.666x^2^-0.182x^3^	0.038	0.614	Constant
Eye	713,150	y=43.690+2.087x-0.595x^2^	0.701	0.004	Increasing
Circulatory system	2,354,977	y=138.002+1.719x-0.969x^2^	0.359	0.088	Constant
Digestive system	6,426,105	y=367.87-4.134x-1.203x^2^	0.576	0.017	Decreasing
Musculoskeletal system	6,289,449	y=360.956+1.452x-1.439x^2^	0.104	0.396	Constant
Genitourinary system	4,536,609	y=254.882-9.823x	0.934	<0.01	Decreasing
Breast	356,194	y=20.869-0.958x-0.124x^2^	0.812	<0.01	Decreasing
Obstetric	8,583,315	y=499.090-0.903x-2.914x^2^	0.014	0.756	Constant
Thoracic	449,447	y=25.808+0.676x-0.116x^2^	0.789	0.001	Increasing
Reconstructive	597,136	y=34.945-1.442x-0.210x^2^	0.890	<0.01	Decreasing
Buco-maxillofacial	156,488	y=6.864-1.72x+0.307x^2^	0.696	0.005	Decreasing
Other surgeries	3,145,864	y=173.918+18.738x	0.938	<0.01	Increasing
Oncology surgery	930,097	y=50.171+2.74x+0.227x^2^	0.798	0.001	Increasing
Total^||^	37,565,785	y=21.69+0.081x-0.104x^2^	0.084	0.449	Constant

*Source: Ministry of Health, Department of Informatics of the Unified Health System, 2017; †Constant 1000; ‡R^2^= Coefficient of determination; §p-value<0.05= Significant trend; ||Constant 100,000.

Overall, the mean hospital stay was 3.8 days. The highest mean length of stay (9.5 days) was in thoracic surgery. In contrast, eye surgeries had the shortest length of stay (0.6 days). In the trend analysis, the mean permanence was decreasing and significant (p <0.01). When analyzed by subgroups, the trend was increasing and significant (p <0.05) in head and neck surgeries, musculoskeletal and obstetric surgeries. With the exception of reconstructive and breast surgery that showed a constant trend, the other subgroups showed a significant decreasing tendency (p <0.05), as shown in Table 2.

**Table 2:** Temporal trend of the average hospital stay according to subgroup of surgical procedures. Brazil, 2008 to 2016*

Subgroup	Length of stay	Model	R^2†^	*p* ^‡^	Trend
Minor surgeries	2	y=2.011-0.088x	0.957	<0.01	Decreasing
Endocrine glands	3	y=2.988-0.051x	0.847	<0.01	Decreasing
Nervous system	8.9	y=8.911-0.116x	0.918	<0.01	Decreasing
Head and neck	3.4	y=3.394+0.051x-0.002x^2^-0.00x^3^	0.768	0.001	Increasing
Eye	0.6	y=0.578-0.055+0.009x^2^	0.816	<0.01	Decreasing
Circulatory system	4.9	y=4.944-0.055x	0.897	<0.01	Decreasing
Digestive system	3.7	y=3.692-0.033x-0.002x^2^	0.882	<0.01	Decreasing
Musculoskeletal system	4.5	y=4.589+0.046x-0.006x^2^-0.001x^3^	0.656	0.008	Increasing
Genitourinary system	2.4	y=2.386-0.028x-0.002x^2^	0.802	0.001	Decreasing
Breast	1.7	y=1.720+0.005x+0.001x^2^-0.001x^3^	0.300	0.126	Constant
Obstetric	2.6	y=2.597+0.004x+0.003x^2^+0.000x^3^	0.525	0.027	Increasing
Thoracic	9.5	y=9.544-0121x	0.942	<0.01	Decreasing
Reconstructive	4.9	y=4.714-0.084x+0.024x^2^+0.006x^3^	0.036	0.624	Constant
Buco-maxillofacial	2.2	y=1.440-0.475x+0.045x^2^+0.015x^3^	0.757	0.002	Decreasing
Other surgeries	6	y=6.1-0.065x	0.905	<0.01	Decreasing
Oncology surgery	4.4	y=4.466-0.18x	0.981	<0.01	Decreasing
Total	3.8	y=4.034-0.064x	0.956	<0.01	Decreasing

*Source: Ministry of Health, Department of Informatics of the Unified Health System, 2017; †R^2^= Coefficient of determination; ‡p-value<0.05= Significant trend.

Overall, the average cost of admission was $ 389.16.

The highest average cost ($ 1,506.26) was in circulatory surgeries. In contrast, the minor surgeries subgroup had the lowest average cost ($ 101.28). The temporal evolution of the mean cost of surgical admissions increased significantly (p <0.01). With the exception of the subgroups that presented a constant trend (minor surgeries and nervous system) and buco-maxillofacial surgery, which had significant decline (p <0.05), in the others, the tendency was shown to be increasing and significant, according to Table 3.

**Table 3:** Temporal trend of the average cost according to the subgroup of surgical procedures. Brazil, 2008 to 2016*

Subgroup	Average cost^†^	Model	R^2‡^	*p* ^§^	Trend
Minor surgeries	101.28	y=369.257+0.201x-1.635x^2^+0.181x^3^	0.212	0.211	Constant
Endocrine glands	168.07	y=603.980+16.404x-1.328x^2^	0.800	0.001	Increasing
Nervous system	979.42	y=3540.228-31.566x-11.661x^2^+4.857x^3^	0.184	0.248	Constant
Head and neck	404.12	y=1473.314+66.095x-9.402x^2^	0.777	0.001	Increasing
Eye	256.05	y=920.373+29.912x-4.377x^2^	0.837	0.01	Increasing
Circulatory system	1.506.26	y=5474.047+142.94x-26.032x^2^	0.824	<0.01	Increasing
Digestive system	256.03	y=930.164+30.516-3.733x^2^	0.834	<0.01	Increasing
Musculoskeletal system	285.41	y=1005.951+29.640x	0.932	<0.01	Increasing
Genitourinary system	146.95	y=538.102+18.049x-2.241x^2^	0.811	<0.01	Increasing
Breast	132.16	y=471.092+14.789x	0.950	<0.01	Increasing
Obstetric	186.85	Y=660.197+11.637	0.902	<0.01	Increasing
Thoracic	729.74	y=2666.978+112.691x-17.654x^2^	0.843	<0.01	Increasing
Reconstructive	364.14	y=1351.301+43.013x-8.355x^2^	0.746	0.002	Increasing
Buco-maxillofacial	167.34	y=474.504-92.479x+5.493x^2^+4.101x^3^	0.600	0.014	Decreasing
Other surgeries	791.29	y=2637.426+194.139x	0.950	<0.01	Increasing
Oncology surgery	788.56	y=2582.467+245.370x+10.593x^2^	0.752	0.002	Increasing
Total	389.16	y=1367.276+72.986x	0.960	<0.01	Increasing

*Source: Ministry of Health, Department of Informatics of the Unified Health System, 2017; †US Dollar quotation on 05/10/2018 = 3.54 reais; ‡R^2^= Coefficient of determination; §p-value<0.05= Significant trend.

Overall, the mortality rate was 1.63%. The highest rate was identified in thoracic surgeries (11.87%) and the lowest (0.02%) in eye surgeries. The temporal evolution of surgical mortality was increasing and significant (p <0.01). In a subgroup analysis, a similar result was obtained in the musculoskeletal, genitourinary and thoracic surgery (p <0.01). In contrast, surgical mortality tended to decline with a significant difference in minor surgeries, nervous system, digestive, obstetric and other surgeries, according to Table 4.

**Table 4:** Temporal trend of mortality according to subgroups of surgical procedures. Brazil, 2008 to 2016*

Subgroup	Mortality rate	Model	R^2†^	*p* ^‡^	Trend
Minor surgeries	0.18	y=0.154-0.017x+0.003x^2^	0.828	<0.01	Decreasing
Endocrine glands	0.19	y=0.222-0.001x-0.004x^2^+0.000x^3^	0.008	0.809	Constant
Nervous system	9.63	y=9.643-0.186x	0975	<0.01	Decreasing
Head and neck	3.98	y=4.070-0.070x-0.012x^2^+0.004x^3^	0.043	0.588	Constant
Eye	0.02	y=0.017-0.003x+0.001x^2^	0.366	0.084	Constant
Circulatory system	3.08	y=3.0440.022x+0.006x^2^+0.000x^3^	0.346	0.095	Decreasing
Digestive system	2.13	y=2.134-0.016x-0.000x^2^+0.000x^3^	0.655	0.007	Decreasing
Musculoskeletal system	0.89	y=0.896+0.016x-0.001x^2^	0.850	<0.01	Increasing
Genitourinary system	0.28	y=0.277+0.013x	0.942	<0.01	Increasing
Breast	0.04	y=0.035+0004x+0.000x^2^-0.000x^3^	0.003	0.888	Constant
Obstetric	0.06	y=0.040-0.008x+0.003x^2^-7.575x^3^	0.549	0.022	Decreasing
Thoracic	11.87	y=11.793+0.242x	0.974	<0.01	Increasing
Reconstructive	1.47	y=1.456-0.029x+0.002x^2^+0.001x^3^	0.289	0.135	Constant
Buco-maxillofacial	0.1	y=0.081-0.003x+0.002x^2^+0.000x^3^	0.188	0.242	Constant
Other surgeries	3.98	y=4.036-0.045x-0.003x^2^	0.701	0.004	Decreasing
Oncology surgery	1.96	y=1.980-0.055x-0.001x^2^+0.002x^3^	0.380	0.077	Constant
Total	1.63	y=1.62+0.024x	0.919	<0.01	Increasing

*Source: Ministry of Health, Department of Informatics of the Unified Health System, 2017; †R^2^= Coefficient of determination; ‡p-value<0.05= Significant trend.

**Figure 1:**
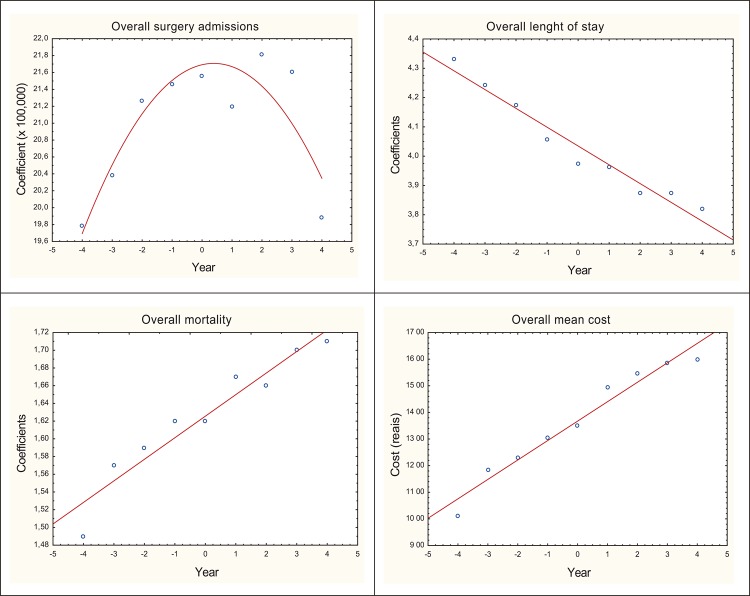
Dispersion diagram of surgical admissions, length of stay, cost and mortality. Brazil, 2008 to 2016

The dispersion diagram showed that the time evolution of surgical admissions according to subgroups of procedures was constant. The average hospital stay decreased, the average cost and mortality increased. The variables length of stay, average cost and mortality presented high coefficients of determination, respectively R^2^=0.956, R^2^=0.919 and R^2^=0.960, establishing a positive and near perfect correlation between mean hospital stay, coefficient of surgical mortality and average cost of admission in relation to time, according to Figure 1.

## Discussion

In this study, during the period of nine years (2008-2016), the time trend of surgical admission according to subgroups of procedures, was constant (p = 0.449). The mean length of stay (3.8 days) decreased (p <0.01), and the mean cost ($ 389.16) and mortality (1.63%) increased (p <0.01). Comparatively, except for the trend in the number of surgical admissions, these results are equivalent to those found in a pioneer and similar study conducted in Brazil between 1995 and 2007. In thirteen years, the trend of surgical admissions (p = 0.012), costs ($ 445.24) and surgical mortality (1.60%) was increasing and significant, and the mean length of stay (4.13 days) was decreasing (p = 0.001)^(^ ^5^ ^)^.

More recently, a study analyzed data on surgical admissions in Brazil in 2014. In this research, the authors evaluated the surgical volume according to the indicators proposed by the Lancet Commission and obtained the rate of 4,433 procedures/100,000 inhabitants/year^(^ ^8^ ^)^. This surgical volume was lower than the goal of 5,000 procedures/100,000 inhabitants/year established by the Commission to guarantee access to essential surgical and anesthetic care when necessary by the population^(^ ^4^ ^)^. Additionally, the average length of stay was 3.6 days and the mortality rate was 1.71%^(^ ^8^ ^)^.

Despite estimates of significant growth in demand for surgery^(^ ^1^ ^)^, this study showed that the temporal trend of surgical admissions by subgroups of procedures remained stationary. In addition, when each subgroup of procedure was analyzed, there was a decreasing or constant trend. In view of these findings, it is recognized that the surgical volume in Brazil, because it remains constant, falls short of the international guidelines that recommend the definition of strategies to increase the access and coverage of the surgical procedures considered essential^(^ ^2^ ^,^ ^4^ ^)^.

In addition, universal access and coverage to health are not satisfactorily guaranteed. Often, when demand is greater than the capacity of the public system, there is inadequacy of public financing for health. This situation constitutes one of the main problems of public health systems, being a permanent source of political and social discontent^(^ ^13^ ^)^.

Despite the progress made by the SUS in improving access to health care for a significant portion of the Brazilian population, it is still a developing health system that continues to strive to ensure universal and equitable coverage^(^ ^14^ ^)^.

A systematic review conducted in 2017 to analyze the effect of economic decline in the countries on surgical volume found that there was a reduction in surgical volume when economic indicators decreased, both in elective and non-elective surgeries^(^ ^15^ ^)^.

In the Brazilian context, despite the approximate four-fold increase in federal funding, since the beginning of the last decade, the federal budget for the health sector has not grown, which has culminated in financing, infrastructure and human resources constraints. In addition, other challenges arise due to changes in the demographic and epidemiological characteristics of the Brazilian population. In order to overcome the challenges faced by the Brazilian health system, a new financial structure and a revision of public-private relations are crucial. Therefore, one of the major challenges faced by SUS is of political order^(^ ^14^ ^)^. Financing, public-private articulation and persistent inequalities cannot be solved solely in the technical sphere. The legal, normative and operational bases have been established. From now on, it is necessary to guarantee to the SUS its political, economic, scientific and technological sustainability^(^ ^14^ ^)^.

Regarding the subgroups of procedures in the different specialties, it was evidenced that in the thoracic surgery, the variables hospital stay and mortality rate presented the highest figures. A study carried out to evaluate the surgical mortality rate in the thoracic surgery department in a hospital in Porto Alegre found that thoracic surgery had a mortality rate that was considerably higher than the total surgical mortality. In 2013, the overall surgical mortality rate was 2.62%, while in the thoracic surgery it was 9.22%. The authors suggested that most surgeries in this specialty is performed in patients with a wide variety of severe conditions, with high risk and emergency ratings that increase surgical morbidity and mortality^(^ ^16^ ^)^.

The subgroup of circulatory surgeries had the highest average cost of hospitalization (US$ 1,506.26). Importantly, the aggravation of many cardiovascular diseases that will require surgical treatment could have been avoided with investment at the primary level^(^ ^17^ ^)^.

This is an important aspect to be considered in the elaboration of health policies, planning and management, since investments in health promotion can reduce the population’s demand to more complex interventions in health services, resulting from acute events that require care in special units that deal with coronary care or intensive care. In addition, myocardial revascularization surgery presents itself as a therapeutic resource to treat complications and advanced stages of the disease. However, these complex procedures require health workers with specific skills, high technology equipment, expensive treatment and tertiary hospital infrastructure^(^ ^17^ ^)^.

Regarding surgical admissions in the subgroup of the circulatory system, a constant temporal trend was observed. This result contrasts with those obtained in a study conducted to evaluate the trend of procedures and mortality related to cardiovascular surgeries performed at the Heart Institute between 1984 and 2007. In that study, the evolution of cardiovascular surgery was increasing^(^ ^18^ ^)^.

Eye, thoracic, and oncological surgeries, as well as other surgeries, showed a growing and significant temporal trend. Considering these results, it can be inferred that, in general, this increasing trend is related to the change in demographic profile of the Brazilian population, since many chronic diseases arising from aging can be treated surgically, such as cataract and oncological diseases.

Among the eye surgeries, the procedure of phacoemulsification with implantation of foldable intraocular lens was the most accomplished (187,265 surgeries), which corresponded to 26.2% of the total eye surgeries performed in the period. It is noteworthy that this surgical procedure is indicated in cases of cataract. This disease is the leading cause of blindness in the world, although it is recoverable by relatively simple and inexpensive surgical intervention that improves the quality of life of individuals and impacts socially. However, estimates show that Brazil is unable to perform the number of cataract surgeries necessary to compensate for the emergence of new cases^(^ ^19^ ^)^.

In an attempt to improve access to cataract surgery, in 1998, the National Cataract Campaign was launched. The Cataract Project aimed to reduce the difficulties of access to ophthalmological assistance, exams and surgery by the population throughout the country. In order to make the project feasible, the federal government determined the guarantee of the financing of all the surgeries performed. However, in 2006, the transfer of the resource was interrupted and its continuity was discouraged^(^ ^19^ ^)^.

Regarding oncological surgeries, of the 15.2 million new cases of cancer in 2015, more than 80% needed surgery. In the world, by 2030, it is estimated that, annually, there will be a need of 45 million surgical procedures^(^ ^20^ ^)^.

Despite advances in the field of radiotherapy and chemotherapy, surgery is important in the prevention, diagnosis, curative treatment, treatment support measures, palliative treatment and reconstruction. In this sense, surgery is considered vital for the reduction of premature mortality due to cancer^(^ ^21^ ^)^. However, globally, less than 25% of cancer patients receive safe, accessible or timely surgery^(^ ^20^ ^)^. In this sense, in Brazil, only 9% of the total resources allocated to oncology are assigned to cancer surgery^(^ ^22^ ^)^.

In conducting this study, some limitations should be considered. In the system, the secondary data obtained may be underreported and contribute to information bias, since the variable surgical admissions included the paid admissions, but did not cover all those performed effectively by the SUS due to the limits defined in the physical and financial programming of the SUS. Likewise, admissions in hospitals with no link with SUS were not considered.

However, we believe that this study is a precursor in the production of knowledge about surgical admissions by SUS in the last decade with national coverage and that the generated evidence can contribute to filling the knowledge gap and scientific advance in this area.

Regarding the implications for the area of health and nursing, the scarcity of available evidence on the epidemiological aspects and trends of surgical admissions and surgeries by specialties in the national context^(^ ^5^ ^,^ ^8^ ^)^ entails a vast field for the development of future research, since knowledge of these trends can be useful for the management, planning and distribution of resources for the health area^(^ ^15^ ^)^.

It is believed that the knowledge produced on the epidemiological data of surgeries performed in each country and its progression over the years is essential for defining strategies and priorities in public health policies^(^ ^5^ ^)^. In the field of nursing, the nurse has the potential to assume a differentiated position in the management of health systems and contributes to the implementation and maintenance of health policies. However, it is still necessary to build and consolidate expressive insertion in decision-making levels in management spaces. For this purpose, changes and investments in several fronts are crucial, such as the development of political, technical and relational competences in the process of training future professionals; the permanent education of nurses working in the labor market; the participation of category organizations focused on the appreciation of professionals in the health system scenario, as well as in the participation in decision-making environments of the different levels of management; and the construction of partnerships with health professionals, users and institutions for the valorization of health, as a citizenship right ^(^ ^23^ ^)^.

## Conclusion

In Brazil, it was evidenced that the trend of 37,565,785 surgical admissions analyzed according to subgroups of surgical procedures, from 2008 to 2016, was constant (p = 0,449). The mean length of stay (3.8 days) decreased (p <0.01), whereas the mean cost ($ 389.16) and mortality (1.63%) increased (p <0.01).

The temporal evolution of the surgeries was increasing and significant in the subgroups of the eye, thoracic, oncological surgeries and other surgeries. Surgeries of the endocrine, digestive, genitourinary, breast, reconstructive and buco-maxillofacial tissues decreased. The others were constant.

Therefore, in nine years, the temporal trend of surgeries remained stable, which is contrary to the international recommendations to increase surgical volume in the countries and guarantee access to surgery. It is believed that these results can support the knowledge of the epidemiological profile of surgical admissions and their temporal evolution in the last decade and contribute to the efficient elaboration of public policies, planning and management towards universal coverage in surgical care.
